# Emergency Medicine Specialists’ Understanding of Stressful Conditions and Coping strategies: A qualitative content analysis

**DOI:** 10.34172/aim.35116

**Published:** 2026-02-01

**Authors:** Hossein Ardalan, Behzad Zohrevandi, Venus Valiani, Nazanin Noori Roodsari, Leila Kouchakinejad-Eramsadati, Naema Khodadadi-Hassankiadeh

**Affiliations:** ^1^Guilan Road Trauma Research Center, Trauma Institute, Guilan University of Medical Sciences, Rasht, Iran; ^2^Internal Medicine Department, Rasoul Akram Hospital, Iran University of Medical Sciences, Tehran, Iran; ^3^Department Emergency Medicine, Poursina Clinical Research Development Unit, Poursina Hospital, School of Medicine, Guilan University of Medical Sciences, Rasht, Iran; ^4^Social Determinants of Health Research Center, Trauma Institute, Guilan University of Medical Sciences, Rasht, Iran

**Keywords:** Coping strategies, Emergency medicine, Qualitative, Stress, Stressor

## Abstract

**Introduction:**

The stressors in emergency medicine and the coping strategies that can help decrease stress have not been well understood. Therefore, our aim was to explain to emergency medicine specialists the perceived stressors and useful coping strategies in the emergency departments in 2024.

**Methods:**

The present study is a conventional qualitative content analysis with an inductive approach. Data were collected using open-ended, semi-structured individual interviews. The participants were emergency medicine specialists of all medical universities in the country. The contents were analyzed using the Graham and Longman content analysis method.

**Results:**

Of 19 participants, the majority were in the 40- to 49-year age group, married, and male. The majority were assistant professors and associate professors of medicine with 10 to 19 years of work experience, who underwent virtual interviews. The participants’ statements revealed three stressor categories with 15 subcategories: individual stressors, concurrent stressors, and collateral stressors. In addition, three stress-reducing coping strategies categories with 15 subcategories were extracted: active individual adaptation, treatment optimization, and receiving support.

**Conclusion:**

The medical field is inherently stressful, but not all stressors faced by medical professionals are individual in nature. Some stressors occur simultaneously and laterally that are not individual but organizational and environmental. The coping strategies explained were active personal adaptation, optimizing treatment, and receiving support. In other words, in addition to the need for medicine to actively resolve damaging stressors with individual self-care, medicine needs comprehensive internal and external support.

## Introduction

 Emergency medicine represents a distinctively high-pressure field characterized by the unpredictability of cases that require rapid and accurate interventions, as well as the execution of intricate procedures.^1,2^ Previous research has shown that 22% of emergency workers had PTSD,^3^ 19.3% experienced burnout and 21.7% suffered from job strain.^4^ In developed countries, the prevalence of depression in these workers has been reported to be between 21.53% and 32.7%.^5-7^

 Certain stressors in emergency rooms, such as child death or abuse,^8^ workplace violence, dealing with infections, crisis management, medico-legal issues, lack of professional connections, disasters and mass incidents and ill or dying family member or friend,^9^ cases requiring high precision, inability to provide ideal care, crowding and noise, heavy workload with low skills and inadequate resources, are particularly distressing, leading to significant discomfort and imbalance among healthcare professionals.^10^

 Chronic stress may culminate in burnout, characterized by emotional fatigue and feelings of inadequacy, especially among emergency physicians. This phenomenon is associated with adverse consequences, including apathy and a heightened risk of mortality.^1,11,12^ Work-related stress is a leading cause of absenteeism, frequent shift changes, and early retirement in the medical workforce.^4,11,13^

 Stress coping strategies refer to a person’s set of cognitive and behavioral efforts to manage and reduce the effects of stress.^14,15^ Although coping strategies are considered positive or helpful, some coping strategies are maladaptive or negative, such as self-blame or escape. Using some negative coping strategies can be dangerous. The most negative coping strategies used by emergency staff were: ignoring and minimizing the problem, excessive worry, anger, self-blame, pessimistic thinking, insularity, resignation, resorting to alcohol, using oral stress-reducing drugs, suicide, and other high-risk behaviors.^16-18^ Similarly, an Iranian study reported that emergency physicians in stressful situations showed negative reactions to reduce stress, the most important of which were withdrawal, frustration and despair, irrationality, agitation, and psychosis.^19^ While employing effective coping strategies is crucial for mitigating occupational stress, current literature appears to have insufficient evidence on the approaches taken by emergency medicine physicians.^20^ Assessing the effectiveness of coping strategies is crucial for enhancing the work environment for emergency department physicians and can guide managers in selecting the best evidence-based approaches to implement.^21^

 A literature review indicates that while various studies have recognized a range of stressors, it remains unclear how much individual factors are distinct from environmental stressors, and besides a few focused on specific cases in emergency medicine, most have concentrated on general working conditions in the emergency settings.^1,12^ Studying stress alongside coping strategies is essential, especially in high-pressure environments like emergency rooms, where doctors and nurses commonly rely on methods such as maintaining a balanced life, problem-solving, drawing from past experiences, managing situations, and gathering information.^22^

 Previous studies on stressors and coping strategies of these specialists have been few. Thus, a qualitative study could effectively address these gaps by exploring medical professionals’ perspectives on occupational stressors and coping strategies to clarify findings from previous quantitative research. Therefore, the aim of the present study was to explain emergency medicine specialists’ perceived individual stressors and coping strategies in the emergency departments of medical universities in the country in 2024.

## Materials and Methods

###  Setting

 After receiving the Code of Ethics (IR.GUMS.REC.1402.166) on June 14, 2023, the present study was conducted in the administrative and virtual environments of medical universities across the country.

###  Design

 The present study is a qualitative content analysis conducted using a conventional and an inductive approach. Content analysis is a suitable method for explaining the perceptions of participants. This method is a systematic and objective means of providing a qualitative and quantitative description of a phenomenon.^23,24^

###  Participants

 Inclusion criteria: Emergency medicine specialists from the country’s medical universities.

 Exclusion criteria: Individuals who, for any reason, left the interview halfway and left the interview location, or later requested that the content of the interview not be included in the analysis. Only one emergency medicine specialist withdrew from the study.

###  Procedures

 The participating units were identified purposefully. In fact, those participants were selected who could provide the information needed to address the study’s objectives.^25^ The sample selection process was conducted based on the principle of maximum diversity, aiming to encompass a wide range of participants across various age groups, marital, genders and work experience. This approach was intended to capture a broad spectrum of experiences. The method employed for this purpose was Snowball sampling. Initially, an interviewee was selected, who was subsequently asked to refer colleagues working in medical centers affiliated with universities of medical sciences throughout the country. Additionally, emergency medicine specialists recommended by the interviewee were also invited to participate in the interviews. The scheduling of interviews was arranged according to the preferences of the participants. Sampling continued until information saturation was achieved, meaning that no new categories or subcategories emerged.^26,27^ In this study, purposive sampling was employed to recruit participants who possessed relevant lived experience and were able to provide rich and meaningful data aligned with the study objectives. Data collection and analysis were conducted concurrently and continued until data saturation was achieved, defined as the point at which no new codes, categories, or subcategories emerged. Data saturation was reached in the seventeenth interview. To ensure the stability of saturation and to enhance the rigor, credibility, and trustworthiness of the qualitative analysis, two additional interviews were conducted. These interviews did not yield any new concepts and served to confirm the established findings. Accordingly, the sampling process was finalized with a total of 19 participants. Of the 19 participants, recruited from various medical universities across the country, the distribution across institutions was as follows: Guilan Medical Sciences University (n = 9), Tehran University (n = 2), Shahid Beheshti University (n = 1), Iran University (n = 1), Isfahan University (n = 2), Arak University (n = 2), Sari University (n = 1), and the Islamic Republic Army University (n = 1). Of the interviews conducted, 11 were held virtually via WhatsApp, as per the interviewees’ requests, citing time constraints and work commitments. Additionally, 8 interviews were conducted face to face, ensuring complete privacy as per the participants’ preferences.

###  Data Collection Tools

 The data for this study were gathered through individual interviews and responses to semi-structured questions. Initially, the research team developed a set of semi-structured questions, which were organized into a preliminary interview guide (Table 1) during an expert panel discussion. This guide was subsequently tested in the first three interviews, serving as a pilot study to identify overarching themes. Based on insight gained from these initial interviews, the guide was revised and expanded. The remaining interviews were then conducted utilizing the refined guide that emerged from the pilot phase. The interviewer, an emergency medicine specialist, carried out the three pilot interviews under the supervision of a professional experienced in qualitative studies, thereby gaining practical experience in administering the interviews. In this study, the participants acquired skills in formulating probing questions and effectively utilizing interrogative words such as who, when, why, and how, as well as the phrase “Can you please explain more?” to deepen their understanding of new concepts and enhance data saturation. At the outset of each session, the interviewer outlined the study’s objectives and methodologies, securing verbal consent from participants, who were informed of their right to withdraw from the study at any point should they choose not to continue. Upon expressing their willingness to participate, demographic information regarding age and work experience was collected as an introductory measure. Subsequently, the interview guide questions were posed. Prior to commencing each session, participants were notified about the audio recording process.

**Table 1:** Interview Guidelines Prepared Based on Expert Opinion (8 People)

**Titles**	**Questions**
Introduction	I am Dr. H A, currently serving as a general practitioner while also pursuing my residency in emergency medicine. It is widely recognized that emergency medicine ranks among the most demanding specialties within the medical profession. Prolonged exposure to stress in this field can result in burnout, which poses significant risks to both practitioners and patients. Consequently, I aim to investigate the experiences of esteemed colleagues and professors in this discipline for my thesis, focusing on the various stressors encountered and the coping strategies employed. I would greatly appreciate the opportunity to gain insight from your perspectives through an open and confidential interview.
General questions	Please introduce yourself and, if desired, describe your work and educational background.
Stressor Questions	1- Describe the typical stressors encountered during your tenure in the emergency department? 2- Discuss the particularly stressful cases or procedures you have encountered in the emergency department to date. 3- Please provide a ranking of the stressors that you and your colleagues recognize in the emergency department, ordered from the most severe to the least severe.
Coping strategies questions	1 - Describe the methods you have used so far that have been able to reduce your job stress. 2- What coping methods do you recommend for reducing stress? 3- What can others do to help reduce your stress?
Final questions	1- What points do you have in mind regarding the purpose of this study that are not mentioned in these questions? 2-If possible, I would like to have your contact number so that if I encounter any ambiguities in the issues discussed, I can resolve them with your help. 3-If you know someone who can help us with this study, please introduce them.

###  Trustworthiness

 For the purpose of examining validity and robustness, we used the four criteria of credibility, dependability, confirm ability, and transferability of Guba and Lincoln.^28^ Participant review was employed to validate the accuracy of the data collected. Specifically, all prepared content was shared with each participant, allowing them the opportunity to review and clarify any ambiguities present. To establish trustworthiness, the data was also presented to additional qualitative researchers, ensuring that they arrived at similar findings and classifications, thereby achieving consensus. For the purpose of verifiability, meticulous documentation of all research phases was maintained, enabling other researchers to trace the data and processes involved. This approach helped to mitigate any potential biases throughout the research. Furthermore, the researchers enhanced transferability by including relevant quotations from participants corresponding to each subcategory and the overarching category.

###  Data Analysis

 Qualitative content analysis was used to analyze the data according to the steps of Granham and Landman.^29^ Following each interview, the researchers meticulously transcribed the audio recordings verbatim after multiple listening. During this phase, the supervisors reviewed the transcripts several times to clarify any uncertainties. Subsequently, the supervisor coded the first interview in the presence of the student. The student then undertook the coding of the remaining interviews, with the supervisor providing oversight. The initial categorization was established based on the codes derived from the first interview, which underwent several iterations of naming until a designation was chosen that encompassed all subcategories. Concurrently, the codes were refined repeatedly, ensuring that any codes incorrectly assigned to a subcategory were reassigned to their appropriate categories. As the interviews progressed and new codes emerged, the necessity for additional subcategories became apparent. This iterative process persisted until data saturation was achieved.

## Results

 The demographic characteristics of the participants are summarized in Table 2. As the Table shows, the majority of participants were in the age group of 40 to 49 years (12 people), married (17 people), and male (11 people). The majority were assistant professors (7 people) and associate professors of medicine (7 people) and had 10 to 19 years of work experience (10 people), and the majority of interviews (11 people) were conducted virtually (over the phone) (Table 2).

**Table 2:** Demographic Characteristics of Participants

**Age**	**Marital Status**	**Sex**	**Work experience**	**Interview method**	**Interview time**
33	Divorced	Male	7 year	Face to face	55min
37	Married	Female	10 year	Face to face	46min
41	Married	Female	12 year	Face to face	47min
47	Married	Female	23 year	Virtual	50min
33	Married	Male	7year	Virtual	45min
47	Married	Male	14year	Face to face	48min
48	Married	Male	19 year	Virtual	50min
45	Married	Female	12 year	Face to face	45min
57	Married	Male	30 year	Face to face	49min
57	Married	Male	33 year	Virtual	48min
39	Single	Female	10 year	Face to face	49min
46	Married	Female	17 year	Virtual	55min
48	Married	Male	18 year	Face to face	48min
48	Married	Male	18 year	Virtual	50min
48	Married	Male	16 year	Virtual	50min
55	Married	Male	33 year	Virtual	47min
46	Married	Male	20 year	Virtual	48min
44	Married	Female	10 year	Virtual	53min
47	Married	Male	18 year	Virtual	50min

 In the fifteenth interview, codes reached saturation, but four more interviews were conducted to ensure saturation, but no new codes were extracted. This is because a qualitative researcher should not stop collecting data if he does not obtain any new information and should seek to select unusual cases to validate and give meaning to the findings.^30^

 As Table 3 show, job stressors from the perspective of the participants consisted of three categories. The first category was individual stressors, which had seven subcategories (Table 3).

**Table 3:** Categories, Subcategories and Important Codes of Occupational Stressors

**Categories**	**Subcategories**	**Important codes**
1. Individual stressors	1.Inability to satisfy physiological requirements	(Medical hypovolemia leads to misdiagnosis 9) (Irritability and drowsiness, not recognizing patients and lack of concentration 10)
	2.Stressful work task	(The most difficult and stressful job in the world 15) (Referring desperate patients from everywhere 18)
	3.Unpredictability of circumstances	(A heart patient passing away in a moment and not being able to do anything for the patient 2) (Sudden deterioration of patients 7)
	4. Risk of Missing the Hidden Sickest Patient	(After 16 years, still afraid of missing a patient in a crowded emergency room 4) (Failure to notice the severity of the patient's condition and waste of time 11)
	5.Challenging procedures	(High failure of intubation 1) (Excessive resuscitation energy 3)
	6.Case criticality	(Cardiac contusion occurring simultaneously with tension pneumothorax in a patient with chest trauma 9) (If the patient develops V-Tach or VF 10)
	7. Anticipatory Anxiety in Critical Future Events	(Feeling insecure in the emergency room workplace 6) (Stress about how to get through a grueling shift and continue 7)
2. Concurrent stressors	1. Congestion	(Stress in the ability to control and manage a busy emergency 12) (Simultaneous treatment of all patients in a very high load 5)
	2. Disputes and Aggression	(Disturbing the emergency atmosphere by agitated, disrespectful companions 11) (Requesting immediate attention for a good-level patient from a doctor who is performing CPR 16)
	3. Absence of adequate services and facilities	(Ambo bag not working during intubation of a sedated patient 3) (lack of empty beds in the emergency room 17)
	4. Team inefficiency	(Assistants not performing the assigned tasks 12) (Not interacting properly with other services or assistants 14)
	5 Bias in triage classification	(Lack of coordination of triage and patient leveling 9) (Severe stress due to the possibility of incorrect triage 1) (In level 1 triage patients, wasting time can lead to patient death 3)
3. Side stressors	1. Economic pressure	(Very low income compared to other medical fields considering the stress they endure 5) (Unfair and inefficient payroll system 6)
	2. Family and community challenges	(Different behavior of patients compared to their behavior in an office or bank 12) (Suffering a spiritual cost of not being with family 15)
	3. Negligence of authorities	(Some medical specialists turning to beauty work 7) (Emergency medicine specialists made great sacrifices during COVID-19, but they were not even given a single sheet of appreciation paper 12)

###  Individual Stressors

####  Inability to Satisfy Physiological Requirements

 Participants identified several stressors as significant contributors to the stress experienced by professionals in emergency medicine (Table 3). Foremost among these is the inability to satisfy physiological needs, which represent the fundamental requirements of human beings according to Maslow’s hierarchy.

 “*Insufficient fluid consumption and nutritional deficiencies can result in critical health situations” (p1). “Prolonged inability to urinate for several hours may lead to decreased water intake by the patient, thereby exerting additional strain on the kidneys and bladder” (p15).*

####  Stressful Work task

 Numerous individuals have asserted that this field is intrinsically stressful, with levels of stress comparable to or potentially exceeding those found in the most demanding professions.

 “*The stress experienced by individuals can be likened to that of a demolition engineer tasked with the critical responsibility of defusing a bomb; a lapse in concentration could result in fatal consequences” (p4). “Similarly, for a gynecologist, the challenge of delivering a baby facing dystocia parallels the intense pressure we feel when attempting to resuscitate an infant in an emergency” (p12).*

####  Unpredictability of Circumstances

 The statements indicated that the primary source of stress for this individual is the unexpected emergence of an event that could not have been anticipated by a healthcare professional.

 “*Heart patients have consistently posed a significant source of stress for me due to the unpredictable nature of their clinical conditions. In an instant, a patient may deteriorate or even succumb, leaving healthcare providers feeling powerless” (p2). “This stress is exacerbated when individuals presenting with seemingly straightforward complaints suddenly experience a decline in their health, often stemming from lack of sufficient information” (p12).*

####  Risk of Missing the Hidden Sickest Patient

 The potential for a patient to become disoriented among individuals who are experiencing well-being has been characterized as genuinely distressing.

 “*The primary source of my ongoing stress is the apprehension that, due to the high volume of patients in the emergency room, individuals who do not exhibit immediate medical concerns may avoid seeking my assistance, resulting in their conditions becoming obscured (p7)”. “The stress associated with these obscured patients was significant until I implemented measures to ensure that their situations did not escalate to morbidity or mortality”(p19).*

####  Challenging procedures

 Furthermore, participants expressed the view that certain procedures, including intubation, presented significant challenges.

 “*The most challenging experience I encountered involved performing a cricothyrotomy on an adolescent in the CPR room” (p10). “Intubating a patient who presented with significant swelling of the tongue and throat poses considerable difficulties, even when utilizing the most advanced medical equipment”(p16).*

####  Case Criticality

 It was also noted that the nature of certain cases can often be inherently intimidating and stressful for any physician.

 “*Patients experiencing myocardial infarction (MI) who are candidates for thrombolytic therapy may necessitate synchronized shock as a result of dysrhythmic tachyarrhythmia”(p19). “Additionally, these individuals may suffer from multiple-trauma stemming from adverse events affecting several critical organs” (p9).*

####  Anticipatory Anxiety in Critical Future Events

 Participants expressed that they harbored apprehensions and anxieties regarding the future, particularly concerning events that have yet to transpire but are perceived as probable (Table 3).

 “*I’m apprehensive about how I’m going to respond while resuscitating a well-functioning patient while they’re expiring” (p1, p16*).

####  Concurrent Stressors

 The second classification of occupational stressors, as perceived by specialists in emergency medicine, is referred to as simultaneous stressors.

####  Congestion

 Participants expressed the view that one of the simultaneous stressors they encountered was the overcrowding in the emergency room, coupled with the pressure associated with implementing life-saving interventions for patients.

 “*A fire that erupted in one of the hospitals within the city unexpectedly led to a significant influx of patients into the emergency department, compounding the existing patient load” (p13). “Following a large-scale altercation, two groups surged into the emergency room, where they continued to engage in physical confrontations, exacerbating the chaotic environment. This overwhelming situation resulted in considerable stress for both myself and my colleagues” (p6).*

####  Disputes and Aggression

 Participants also mentioned that disputes, aggressive behavior, and confrontations with medical professionals and support staff create significant stress, which not only distracts the physician’s focus but also adversely affects patient care.

 “*Disagreements often arise among companions who believe that, regardless of the non-emergency status of their patient’s condition, they are entitled to emergency services” (p12).” Such conflicts between staff and companions can be particularly stressful, as the patient remains without the necessary care” (p15).*

####  Absence of Adequate Services and Facilities

 Absence of adequate emergency facilities, attributed to critical needs, represents another significant source of stress that has been highlighted. In instances involving children, the deficiency of specialized provisions can potentially result in the loss of a child’s life.

 “*The stress was that we had sedated a child, but we didn’t have the right laryngoscope connector so we had sedated him for no reason. The child was sedated unnecessarily (p3).”*

 “*The stress in the context of a minibus-bus collision was the lack of enough equipment for all patients in the emergency department. The limited availability of central venous lines further complicated efforts to stabilize patients’ conditions (p15)”.*

####  Team Inefficiency

 Participants indicated that any interruption in collaborative efforts can lead to increased stress levels among healthcare practitioners.

 “*A nurse retains medical directives without authorization, citing inadequate compensation as a justification (p4)”. *“*In the context of Iran, the absence of collaborative teamwork results in the overwhelming burden of responsibilities being placed solely on the physician” (p17).*

####  Bias in Triage Classification

 The significance of triage in evaluating patients who arrive at the emergency department is explicitly highlighted. Inadequate or excessive triage can lead to patients being directed to inappropriate departments, thereby hindering the timely provision of necessary medical services.

 “*The potential for a nurse to misclassify a patient’s condition during triage can lead to significant stress, particularly when there is a risk that a critically ill patient may not receive the appropriate diagnosis” (p1). “Such errors in triage and classification not only consume valuable time but also jeopardize the patient’s life” (p10) (Table 3).*

####  Side Stressors

 Participants identified certain stressors that indirectly influenced mental engagement and contributed to a decline in concentration among medical professionals. This category encompassed three distinct subcategories.

####  Economic Pressure

 The participants indicated that the financial strain and the living conditions experienced by these professionals posed significant challenges.

 “*Job-related stress is exacerbated by inconsistent and erroneous salary disbursements” (p5).” The financial rewards or income we receive do not align with the expectations set by the volume and complexity of our responsibilities” (p19).*

####  Family and Community Challenges

 The participants expressed that a significant gap in comprehension regarding night shifts and the medical profession existed within their families and communities. This lack of understanding undoubtedly contributed to mental conflicts experienced by the specialists, affecting their overall well-being and professional performance.

 “*The perception of dissatisfaction with medical services, coupled with a lack of respect from the community, can lead to a sense of disappointment among healthcare professionals” (p11).” Without the backing and collaboration of family members, the medical field is likely to encounter significant challenges as time progresses” (p19).*

####  Negligence of Authorities

**Figure 1:**
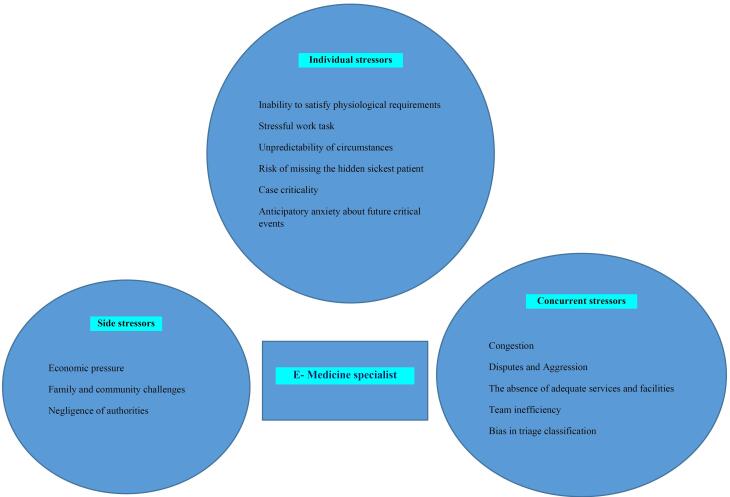
Presentation of Stressors on Emergency Medicine Specialists from the Perspective of the Participants (19 People)

 Participants expressed concerns regarding the lack of attention from authorities towards this sector, highlighting issues such as the inequity and ineffectiveness of the salary structure, inadequate acknowledgment of the field’s dignity and prestige, and insufficient support for enhancing motivation to pursue careers in this area. Additionally, they noted the significant emigration of a considerable number of medical professionals as a critical issue (Table 3). Figure 1 shows the types of stressors that an emergency medicine specialist faces.

 “*The significant absence of a decision to pursue a career in medicine, coupled with the limited number of admissions available in this discipline, creates a profound void. The field of medicine is subject to considerable pressure” (p15).” In Iran, there are no adequate university resources dedicated to medical education, resulting in a restricted number of graduates entering this profession” (p17). *

 The data presented in Table 4 indicates that participants identified four distinct categories of stress-reducing strategies: active personal adaptation, optimization of treatment, seeking support, and maladaptive adaptation.

**Table 4:** Categories, Subcategories and Important Codes of Stress-reducing Strategies

**Categories**	**Subcategories**	**Important codes**
1. Active individual adaptation	1. Obviating basic needs	(Recommendation of drinking healthy fluids and appropriate foods to prevent burn-out 9) (Recommendation of using fluids and staying hydrated in the emergency room 13)
	2. Recreational activities	Pursuing a hobby at least once a week 1) (Recommending going to soccer, horseback riding, etc. 3)
	3. Psychological and spiritual growth	(Not expecting humane behavior from others (to reduce stress 2) (Relying on and seeking help from a supernatural force, for example, God Almighty and the pure Imams 5)
	4. Upskilling	(Need for mastering all the techniques needed to establish an airway 6) (Need for always being up-to-date 13)
2. Treatment optimization	1. Foresight	(Presetting services to serve the patient in the shortest time at the highest quality 6) (Knowing the possibility of adding an extra bed in a critical situation 7)
	2. Quick assignment	(Need for managing time and energy in shifts 13) (Presenting on time at the patient's bedside 5)
	3. Team coordination	(Understanding the teamwork nature of working in the emergency department 2) (Paying attention to the mental and physical conditions of the staff 4)
	4. Peaceful and good interaction	(Finding a common language according to the socio-cultural level of the patient and the companions 3) (Learning the necessary skills to interact better with the companions 5)
3. Receive of support	1. Providing manpower and supplies	(Having two specialists simultaneously, one person in CPR and one person in the emergency room 2, 17)
	2. Training and consulting	(Periodically referring doctors to a psychologist 2) (Need for participating in stress reduction workshops and classes 19)
	3. Improving shift scheduling	(Converting 12-hour shifts to 8-hour shifts by increasing the number of colleagues 16) (Usefulness of considering reducing shift hours to reduce consequences 6)
	4. Good family and friendship relationships	(Safe and calm family environment, a good place to renew one's spirit and strength 7) (Important role of the family in coping with nights and heavy shifts and allowing them to rest 17)
	5. Financing/Social	(Good income to attract students to the field 11) (Raising salaries and allowances 15)
4. Faulty adaptations	1. Extreme thoughts and actions	(Continuous walking in the emergency room and constantly seeing the patient's radiographs and tests out of obsession 2) (Obsession with constantly returning and checking on the patient 3)
	2. Self-harm	(Suicidal thoughts or attempts by residents or physicians 1) (Resorting to smoking and sedatives to relieve mental and psychological conditions 17)

####  Active individual adaptation

 This classification emerged from the coping mechanisms that the medical field employs to tackle its specific stressors. It encompasses four distinct subcategories.

####  Obviating basic needs

 Participants indicated that one effective strategy involved addressing fundamental needs promptly, either at the outset or during designated breaks.

 “*It is advisable to ensure sufficient hydration, particularly through the intake of water, diluted tea, herbal infusions, and fruits” (P14). “The establishment of a formalized and legally binding protocol for medical examinations is essential, as individuals may neglect to prioritize their own health needs” (P13).*

####  Recreational activities

 Participation in leisure pursuits, including travel and various sports like soccer and equestrian activities, was also noted.

 “*Engaging in physical activity should be viewed as beneficial not solely for the purpose of weight management, but also for fostering mental and emotional well-being” (P3).” Taking a brief hiatus from the workplace, even for a mere 48 hours, while refraining from any work-related communications, can significantly enhance one’s overall relaxation and refreshment” (P12).*

####  Psychological and spiritual growth

 Several individuals have expressed the view that addressing stress effectively necessitates a focus on both psychological development and spiritual advancement.

 “*At the commencement of each shift, I step into the emergency room with a sense of reverence, invoking the presence of God and placing my trust in His guidance. In addition, I seek solace and support from spiritual leaders, such as imams, to bolster my resilience” (P4).” To maintain a professional distance, I consciously engage my cognitive faculties to detach from the narratives surrounding the patients, effectively erasing any related details from my short-term memory” (P15).*

####  Upskilling

 Participants expressed that enhancing their ability to manage stress was achievable through the cultivation of their unique specialized skills. This included engaging in targeted training programs, remaining informed about current developments, and utilizing relevant resources for reference.

 It is essential to remain informed about the most recent advancements in scientific research, innovative procedures, management strategies, and the latest methodologies endorsed by the AHA. Typically, when a physician possesses the requisite skills, comprehends the established protocols, and adheres to them appropriately, coupled with the stability of the patient’s ABCD parameters, the physician is likely to experience a reduction in stress levels (Table 4).

####  Treatment optimization

 Participants expressed the view that effective management and optimization of the treatment process could lead to a reduction in certain stressors. This perspective encompassed four distinct subcategories.

 5-1) Foresight

 Participants indicated that stress management can be effectively approached through proactive measures, including the inspection of the emergency trolley and the organization of emergency personnel prior to the commencement of the shift.

 “*Professors emphasize the importance of anticipating potential challenges to prevent the occurrence of medical errors” (p1).* “*A proactive approach involves collaborating with the supervisor at the start of each shift to address staffing issues and ensure that the emergency trolley is adequately stocked with necessary medications” (p15).*

####  Quick assignment

 Furthermore, prompt allocation of patients can alleviate the pressures associated with overcrowding and the potential mismanagement of patients.

 “*Rapid assignment refers to the process of expediting a patient’s discharge in order to facilitate the admission of new patients” (p6). “This swift approach to patient care not only aims to enhance the quality of treatment provided but also promotes the release of endorphins within the body, contributing to a more favorable healing environment” (p10).*

####  Team coordination

 Participants indicated that effective coordination within a team can alleviate work-related stress. This outcome is attainable through the presence of skilled, knowledgeable, and well-trained individuals who possess a comprehensive understanding of collaborative practices in the emergency department.

 “*A well-functioning emergency room is contingent upon the presence of competent secretaries, skilled nurses, and effective managers within the facility” (p2).” To enhance the quality of care provided in an emergency setting and alleviate staff anxiety, it is imperative that there is consistent and accurate collaboration among the team members” (p10).*

####  Peaceful and good interaction

 Participants emphasized the importance of fostering an empathetic rapport with colleagues, patients, and their companions. By maintaining a composed and reassuring presence, medical professionals can effectively mitigate potential disputes and conflicts that may arise among companions.

 “*Addressing a patient’s companion’s aggressive behavior from a medical perspective necessitates a careful and composed approach” (p1).” In situations where a patient’s companion exhibits ingratitude and unreasonably high expectations, it is advisable to maintain emotional detachment and preserve one’s own morale” (p4) (Table 4).*

####  Receive of support

 The statements provided by the participants suggest that a variety of coping strategies are employed when healthcare professionals find themselves in situations where external assistance is necessary to alleviate stress. This encompasses five distinct subcategories.

####  Providing manpower and supplies

 “*Establishing a standardized emergency room is essential, encompassing adequate physical space, sufficient human resources, and an appropriate number of staff members” (p11).” The resuscitation area, along with other emergency facilities, must be outfitted with the requisite equipment to promptly commence effective life-saving interventions “(p19).*

####  Training and consulting

 “*Regular training sessions are essential for healthcare professionals as they provide critical updates on scientific advancements and foster appropriate conduct” (p4).” Engaging in courses focused on communication skills, along with proactively consulting with psychiatrists and psychologists when assistance is required, prove to be highly beneficial” (p16).*

####  Improving shift scheduling

 “*More than 13 to 14 twelve-hour-long shifts can lead to burnout” (p7).” In the world, emergency medicine specialists are expected to work approximately 8 to 12 twelve-hour or eight-hour shifts” (p9).*

####  Good family and friendship relationships

*The family serves a crucial supportive function, providing assistance to the physician (p11). Engaging in discussions about workplace challenges with family members and obtaining their insights can significantly contribute to alleviating stress (p18)*.

####  Financing/Social

 “*There should be a balance between pay and the stressful workload of emergency medicine and the harsh working conditions” (p6).” Incomes should be made such that people are attracted to this field, and fees and rates should be increased” (p11) (Table 4).*

####  Faulty adaptations

 The statements provided by the participants suggest that a specific category of coping strategies is employed in a manner that adversely affects the medical professionals. This category encompasses two distinct subcategories.

####  Extreme thoughts and actions

 “*I find myself perpetually traversing the emergency room, driven by an obsession that compels me to examine the patients’ radiographs and test results” (p2). “My time is predominantly consumed within this environment, engaging with the residents, even in instances when the patients are not physically present in the emergency room” (p12).*

####  Self-harm

 “*Personnel, including residents, have turned to smoking and the use of sedatives as a means to alleviate psychological distress” (p17).” In an effort to combat the exhaustion associated with extended work hours, they often rely on substances that may produce harmful side effects” (p13). (Table 4).*

## Discussion

 The experiences of our participants indicated that they encountered numerous and significant stressors within the field of medicine. In alignment with this finding, Campbell *et al. *noted that nearly 50% of Turkish emergency medical professionals expressed considerable dissatisfaction with their overall quality of life, alongside experiencing moderate levels of burnout and traumatic stress.^31^ The subsequent sections will outline the various categories of stress identified by these professionals, as well as the coping strategies they proposed, supported by relevant concordant and non-concordant studies that provide evidence for each category.

 Medical professionals frequently encounter individual stressors that significantly impact their well-being. Previous research corroborates these observations, as demonstrated by Janicki and colleagues, who investigated the physiological stress levels of emergency room residents. They utilized metrics such as heart rate (HR), heart rate variability (HRV), and indicators of mental stress, revealing that those in emergency medicine endure both acute mental and physiological stress during their clinical duties.^32^ Research has highlighted that depression and sleep-wake disorders represent significant challenges for emergency physicians. A study conducted by Lee *et al.* revealed that a substantial proportion (nearly one-third) of emergency medical residents in South Korea experience issues related to sleep-wake patterns.^33^ The American College of Emergency Physicians also states that the nature of emergency medicine duties is more stressful than other medical specialties. Shift work, scheduling, risk of exposure to infectious diseases, ED violence, malpractice, and compensation are among these. Individual stress reduction strategies were similar to the findings of the present study, such as meeting basic needs during rest, participating in recreational activities, and developing specialized personal skills.^34^ Maghsoudi and his team have previously reported a noteworthy correlation between the practice of gratitude and the alleviation of stress, suggesting that the implementation of gratitude programs could be beneficial in sustaining and enhancing the mental well-being of emergency technicians working in hospital settings.^35^ The individuals involved in this study proposed comparable coping mechanisms for the identified medical stressors. These strategies included addressing fundamental needs, such as ensuring adequate rest and hydration, as well as nourishment during quieter periods in the emergency room. Additionally, they emphasized the importance of engaging in regular physical activity and recreational activities. Participants also highlighted the significance of fostering their mental and spiritual well-being through involvement in religious events, meditation, and yoga practices. Furthermore, they expressed the value of enhancing their self-efficacy in applying these techniques by committing to continuous learning and staying informed.

 Physicians often encounter various stressors that can exacerbate their overall stress levels, particularly when they are faced with individual challenges. A study conducted by Feeks and colleagues examined the prevalence and risk factors associated with burnout among pediatric emergency medicine practitioners, revealing that adverse work conditions were the primary contributors to burnout in this field.^36^ Aronson Moussa *et al. *demonstrated that the stress and anxiety experienced by emergency physicians are influenced by various concurrent factors, including ineffective collaboration with nursing staff and the challenges posed by a lack of available beds.^37^ The significance of education, development of coping strategies, and provision of counseling services were proposed as essential measures to mitigate stress levels among healthcare professionals.^36^ An educational intervention aimed at emergency medicine residents was developed in Chicago. One month before the evaluation, the intervention group underwent training utilizing the mental performance tool known as “breathe, talk, see, and focus”. The findings revealed that the medical professionals regarded this training as significant and beneficial, suggesting that more comprehensive or prolonged programs could be advantageous in alleviating stress.^37^ Emergency medicine is characterized by elevated stress levels and a significant risk of burnout among its practitioners. Therefore, facilitating access to a social worker for professionals in this field may serve as an effective strategy to alleviate stress and enhance their overall well-being.^38^ The current study outlines several effective coping strategies, including the implementation of regular training sessions focused on academic triage techniques, which aim to enhance the efficiency of patient care through the proper education of triage nurses and ongoing assessments. Additionally, fostering team morale through in-group meetings is emphasized, alongside the organization of courses and workshops led by psychiatrists to help manage stress among students and medical faculty. Lastly, the importance of seeking professional counseling when individuals feel the need for support is highlighted as a recommended coping mechanism.

 Side stressors were stressors that were on the periphery of work but caused mental engagement and lack of concentration in medical practice. Regarding the financial pressure and problematic living situation of these specialists, it was previously reported that the monthly income of these doctors in Iran is very low and does not correspond to their heavy workload.^39^ Issues of family and community challenges, such as family’s lack of understanding of night shifts and society’s lack of understanding of the difficulties and stresses of the medical field, were also among the statements of these participants, which caused stress and mental conflict for these professionals. The results of the present study are consistent with the study by Clough and colleagues in 2017.^40^ Research has indicated that physicians experiencing greater interpersonal difficulties with their colleagues tend to report elevated levels of burnout, diminished job satisfaction, increased absenteeism, and a higher likelihood of leaving their careers prematurely or opting for early retirement. In examining the inadequacies of institutional responses to medical challenges, McCormick and colleagues investigated the perceptions of job-related stress and coping mechanisms among emergency room nurses and physicians, highlighting the distinctions between these two professional groups. Their thematic analysis revealed that excessive workloads, coupled with insufficient leadership and management support, significantly contribute to the stress experienced by these healthcare professionals.^41^ Additionally, Moussa and colleagues grounded their conclusions in earlier research, indicating that the stress and anxiety experienced by emergency physicians typically originate from factors external to their work shifts.^42^ In addressing external stressors, Clough and colleagues (2017) proposed various psychological interventions aimed at alleviating stress among physicians.^40^ One notable recommendation is provision of psychological counseling for those physicians who find themselves unable to manage their stress independently, as this can help eliminate detrimental thoughts and behaviors. Additionally, implementing coping mechanisms such as fostering personal resilience, along with organization-centered strategies like leadership development, strategic recruitment, appropriate staffing, and sufficient resource allocation, can effectively mitigate job-related stress and enhance individual coping efforts.^41^ In the current investigation, various coping strategies aimed at facilitating support from others were identified. Within the realm of financial challenges, it was suggested that timely and accurate payment of salaries and allowances is essential to prevent excessive working hours, thereby ensuring a balance between remuneration and the demanding workload characteristic of emergency departments. Additionally, enhancing the compensation for other staff members was noted as a means to foster teamwork and maintain morale. Regarding family and community dynamics, it was emphasized that understanding from family members or partners regarding the necessary time for mental and emotional recuperation post-duty is crucial. Recommendations included fostering a tranquil home environment, allocating quality time for family interactions, prioritizing personal well-being, providing financial assistance for family formation, and promoting a culture of peace among emergency room visitors. In addressing the issue of negligence by officials, it was proposed that authorities should alleviate the financial burdens and living conditions faced by healthcare professionals. By enhancing motivation within this workforce, it is anticipated that there will be a greater inclination to remain in the field, thus mitigating the emigration of medical practitioners. Furthermore, establishment of general hospitals, enhancement of hospital infrastructure, presence of robust security and law enforcement in emergency settings, and availability of resident specialists in non-teaching hospitals were highlighted as critical improvements to strengthen emergency departments both in terms of personnel and resources.

 In the current investigation, maladaptive behaviors such as extreme thoughts, self-harm, and other negative coping strategies were identified. Cildoz and colleagues similarly observed that healthcare workers face elevated levels of substance abuse, depression, and anxiety linked to occupational stress, particularly in emergency medicine, where the unpredictable nature of patient emergencies contributes to a high-stress environment.^43^ Research by Rastgou *et al.* further established a correlation between various coping styles and the psychological vulnerability of emergency medicine specialists. The findings suggest that interventions aimed at reducing dysfunctional attitudes and cognitive distortions, while enhancing cognitive emotion regulation, could significantly mitigate the psychological challenges faced by professionals in this demanding field.^44^ The responsibilities of an emergency medicine specialist are significantly more intricate compared to those of other healthcare professionals. Consequently, factors such as inexperience, vulnerability, or an inability to make sound decisions, along with difficulties in managing emotions and stress, can result in medical errors. In this context, cognitive-behavioral therapy focused on emotion regulation may prove beneficial in enhancing the job satisfaction of emergency medicine specialists while also improving their capacity to manage anger and stress. Research conducted by Forghani *et al*. indicates that such therapeutic interventions enable emergency medicine specialists to better regulate their emotional responses and maintain emotional control during or following high-pressure or distressing situations.^45^ The coping strategies proposed by these researchers align closely with those articulated in the current study. Their suggestions, which include engaging with psychoanalytic podcasts and exploring literature rooted in existentialism, emphasize the notion that the burden of managing human lives and well-being does not rest solely on the medical profession. Additionally, they advocate for the development of skills to manage perfectionism, as well as to address obsessive and intrusive thoughts, encapsulating a range of strategies highlighted by these specialists.

 Based on the research referenced in this discourse, which aligns with the findings of the current study, it can be inferred that the various forms of stress identified among Iranian emergency medicine specialists are also prevalent among diverse groups of physicians globally. Consequently, the strategies proposed by Iranian medical professionals may prove to be effective and beneficial for their counterparts in other regions of the world.

 Stress-reducing coping strategies indicate that professionals in this domain must proactively engage in various areas to fulfill fundamental needs, partake in recreational activities, foster psychological and spiritual development, and enhance their skill sets. Furthermore, by refining treatment approaches and employing techniques such as foresight, prompt task allocation, team collaboration, and empathetic communication, a considerable amount of stress can be alleviated. Support through securing essential resources, along with training and counseling, can lead to improvements in shift scheduling, interpersonal relationships with family and friends, and overall financial and social stability, thereby mitigating life stressors. Additionally, through spiritual and psychological education, maladaptive behaviors, including extreme thoughts, harmful actions, and self-injury, can be effectively prevented.

## Conclusion

 It was found that stress is an inseparable part of the lives of emergency medicines specialists. Therefore, stress management should be included in the self-care plan of each specialist. Psychiatric consultations should be provided in case of symptoms of stress. On the other hand, responsible institutions should provide a comprehensive support system for these specialists.
